# Supplementary material for: A fault-intrusion-tolerant system and deadline-aware algorithm for scheduling scientific workflow in the cloud
Source: PeerJ Comput Sci. 2021 Nov 2;7:e747. doi: 10.7717/peerj-cs.747 (PMC8576554; doi:10.7717/peerj-cs.747)
Supplement: Supplemental Information 6 [file peerj-cs-07-747-s006.zip › Myproject1.1/docs/org/cloudbus/cloudsim/core/package-use.html]

Uses of Package org.cloudbus.cloudsim.core


JavaScript is disabled on your browser.


- Overview
- Package
- Class
- Use
- Tree
- Deprecated
- Index
- Help

- Prev
- Next

- Frames
- No Frames

- All Classes

# Uses of Package org.cloudbus.cloudsim.core

- Packages that use org.cloudbus.cloudsim.core

  | Package | Description |
  | --- | --- |
  |  |  |
  | --- | --- |
  | org.cloudbus.cloudsim |  |
  | org.cloudbus.cloudsim.core |  |
  | org.cloudbus.cloudsim.core.predicates |  |
  | org.cloudbus.cloudsim.examples |  |
  | org.cloudbus.cloudsim.network.datacenter |  |
  | org.cloudbus.cloudsim.power |  |
  | org.workflowsim |  |
- Classes in org.cloudbus.cloudsim.core used by org.cloudbus.cloudsim

  Class and Description

  SimEntity

  This class represents a simulation entity.

  SimEvent

  This class represents a simulation event which is passed between the entities in the simulation.
- Classes in org.cloudbus.cloudsim.core used by org.cloudbus.cloudsim.core

  Class and Description

  DeferredQueue

  This class implements the deferred event queue used by `Simulation`.

  FutureQueue

  This class implements the future event queue used by `Simulation`.

  SimEntity

  This class represents a simulation entity.

  SimEvent

  This class represents a simulation event which is passed between the entities in the simulation.
- Classes in org.cloudbus.cloudsim.core used by org.cloudbus.cloudsim.core.predicates

  Class and Description

  SimEvent

  This class represents a simulation event which is passed between the entities in the simulation.
- Classes in org.cloudbus.cloudsim.core used by org.cloudbus.cloudsim.examples

  Class and Description

  SimEntity

  This class represents a simulation entity.

  SimEvent

  This class represents a simulation event which is passed between the entities in the simulation.
- Classes in org.cloudbus.cloudsim.core used by org.cloudbus.cloudsim.network.datacenter

  Class and Description

  SimEntity

  This class represents a simulation entity.

  SimEvent

  This class represents a simulation event which is passed between the entities in the simulation.
- Classes in org.cloudbus.cloudsim.core used by org.cloudbus.cloudsim.power

  Class and Description

  SimEntity

  This class represents a simulation entity.

  SimEvent

  This class represents a simulation event which is passed between the entities in the simulation.
- Classes in org.cloudbus.cloudsim.core used by org.workflowsim

  Class and Description

  SimEntity

  This class represents a simulation entity.

  SimEvent

  This class represents a simulation event which is passed between the entities in the simulation.

- Overview
- Package
- Class
- Use
- Tree
- Deprecated
- Index
- Help

- Prev
- Next

- Frames
- No Frames

- All Classes
